# The Association between Genetic Polymorphism and the Processing Efficiency of *miR-149* Affects the Prognosis of Patients with Head and Neck Squamous Cell Carcinoma

**DOI:** 10.1371/journal.pone.0051606

**Published:** 2012-12-14

**Authors:** Hsi-Feng Tu, Chung-Ji Liu, Che-Lun Chang, Pei-Wen Wang, Shou-Yen Kao, Cheng-Chieh Yang, En-Hao Yu, Shu-Chun Lin, Kuo-Wei Chang

**Affiliations:** 1 Department of Dentistry, National Yang-Ming University Hospital, I-Lan, Taiwan; 2 Department of Oral and Maxillofacial Surgery, MacKay Memorial Hospital, Taipei, Taiwan; 3 MacKay Medical College, New Taipei City, Taiwan; 4 Department of Stomatology, Taipei Veterans General Hospital, Taipei, Taiwan; 5 Institute of Oral Biology, National Yang-Ming University, Taipei, Taiwan; 6 Department of Dentistry, National Yang-Ming University, Taipei, Taiwan; Geisel School of Medicine at Dartmouth, United States of America

## Abstract

MicroRNAs (miRNAs) play important roles in modulating the neoplastic process of cancers including head and neck squamous cell carcinoma (HNSCC). A genetic polymorphism (rs2292832, C>T) has been recently identified in the precursor of *miR-149*; nevertheless its clinicopathological implications remain obscure. In this study, we showed that *miR-149* is down-regulated in HNSCC compared to normal mucosa and this is associated with a poorer patient survival. In addition, HNSCC patients with the T/T genotype have more advanced tumors and a worse prognosis. Multivariate analysis indicated that patients carried the T/T genotype have a 2.81-fold (95% CI: 1.58–4.97) increased risk of nodal metastasis and 1.66-fold (95% CI: 1.05–2.60) increased risk of mortality compared to other groups. T/T genotype also predicted the worse prognosis of buccal mucosa carcinoma subset of HNSCC. *In vitro* analysis indicated that exogenous *miR-149* expression reduces the migration of HNSCC cells. Moreover, HNSCC cell subclones carrying the pri-*mir-149* sequence containing the T variant show a low processing efficacy when converting the pre-*mir-149* to mature *miR-149*. These findings suggest that *miR-149* suppresses tumor cell mobility, and that the pre-*mir-149* polymorphism may affect the processing of *miR-149*, resulting in a change in the abundance of the mature form miRNA, which, in turn, modulates tumor progression and patient survival.

## Introduction

Mature microRNAs (miRNAs) are small endogenous non-coding RNAs consisting of 20∼22 nucleotide duplexes. After being transcribed, primary miRNAs (pri-mirs) are converted into precursor miRNA (pre-mirs) by Drosha in the nucleus, and then further processed into mature miRNAs by Dicer in the cytosol. The mature miRNAs complex with RISC and regulate target genes by mRNA cleavage through base pairing, by translational repression via binding to the 3′ untranslated region (3′-UTR) of the mRNA, or by deadenylating the mRNA [1]. MiRNAs are very important in terms of biological functioning and disease pathogenesis because one miRNA can target several genes and one gene can be regulated by several miRNAs. Thus a miRNA may play pluripotent roles in the modulation of a range of pathogenic processes [2].

The contribution of miRNAs to human carcinomas, including those of breast cancer, lung cancer, prostate cancer, stomach cancer and other sites, has been published widely [3]–[9]. Head and neck squamous cell carcinoma (HNSCC) includes SCC of oral cavity, oropharynx, hypopharynx and larynx. This disease is the 6^th^ most common cancer worldwide [10]. The high incidence of HNSCC in South Asia countries is highly associated with the synergistic effects of betel quid (BQ) chewing, tobacco smoking, and other carcinogenic substances [11], [12]. MiRNAs also deregulated the genesis and progression of HNSCC. Up-regulation of the oncogenic miRNAs, *miR-184*, *miR-21* and *miR-31*, contributes to HNSCC carcinogenesis by targeting tumor suppressor genes [5], [13], [14]. However, *miR-200c* and *miR-375* have been shown to suppress the invasion or stemness properties in HNSCC [15]. Furthermore, down-regulation of *miR-205* has been found to be associated with the locoregional recurrence of HNSCC [16]. Preliminary screenings also suggested alterations in other miRNAs occur during neoplastic process of HNSCC [5], [14].

Genetic polymorphisms within a miRNA sequence, a target gene sequence or gene sequences associated with miRNA processing are thought to have a functional impact on how miRNAs are active in tumors [17], [18]. A variant allele of the *let-7* miRNA complementary site of the K-ras 3′-UTR has been shown to affect the survival of oral cancer patients [17]. The presence of the homozygous T/T genotype in rs11614913 of pre-*mir-196a*2 has been shown to reduce the survival of pharyngeal SCC patients [19]. An earlier study of ours also showed that the C variant of rs2910164 in pre-*mir-146a* is associated among patients with a worse cancer progression [20]. The rs2292832 C/T polymorphism in pre-*mir-149* has been reported to be associated with an increased risk of gastric carcinoma among males [21]. However, studies also failed to demonstrate the prediction power of this polymorphism when HNSCC and lung carcinoma are examined [22], 23. The combined genotypic polymorphisms found in four pre-mirs, including a pre-*mir-149* polymorphism, have been shown to be associated with an increased risk of HNSCC [22]. However, the predictive role of the rs2292832 polymorphism in pre-*mir-149* with respect to tumor progression, and the activity of this polymorphism in the regulation of *miR-149* expression remain unclear. *miR-149* has been found to be down-regulated in malignancies including renal cell carcinoma, prostate carcinoma and astrocytoma [24]–[26]. Our previous and other’s preliminary studies also demonstrated the down-regulation in oral carcinomas [5], [14]. In this context, the present study’s aim is to investigate the deregulation of *miR-149* during head and neck carcinogenesis and to explore the implications of the pre-*mir-149* polymorphism with respect to HNSCC susceptibility and progression.

**Table 1:** Clinicopathological variables of the subjects.

Variables	HNSCC (*n* = 273)	Control (*n* = 122)
***Age*** (year)		
Mean ± SD	53.0±11.0 years	53.5±13.4 years
Range	27–82 years	21–85 years
***Gender*** (Male : Female)	251∶22	60∶62
***Site***		
Buccal mucosa	119	
Tongue	70	
Gingiva	45	
Retromolar trigone	7	
Mouth floor	7	
Oropharynx	15	
Hypopharynx	1	
Lip	7	
Unspecified	2	
***Tumor size***		
T1–T3	137	
T4	136	
***Neck lymph node metastasis***		
N (−)	173	
N (+)	100	
***TNM staging***		
Stage I–III	121	
Stage IV	152	
***Perineural invasion***		
(−)	244	
(+)	29	
***Lymphovascular invasion***		
(−)	246	
(+)	27	
***Follow-up period*** (Mean ± SD)	50.4±32.7 months	

The tumor staging was according to American Joint Committee on Caner (AJCC) TNM staging system. The T represents tumor size and the N represents lymph node metastasis. T1: tumor size less than 2 cm; T2: tumor size between 2 to 4 cm; T3: tumor size larger than 4 cm; T4: tumor invading to adjacent tissues or spaces.

## Methods and Materials

### Ethics Statement

The study samples were collected after obtaining written informed consent and this study was approved by The Institutional Review Board of National Yang-Ming University Hospital (IRB approval no. 2010A013).

**Table 2:** Primers and amplicons.

**Amplification of pre-mir-149 for genotyping**	
Amplicon	
gtcttcactcccgtgcttgtccgaggagggagggagggacgggggctgtgctggggcagc(c)ggaacaacgcaggtcgccgggccggctgggcgagttggccgggcggggctgaggggtcggcgggggaggctgaggcgcgggggccggtgcgcggccgtgagggggtgtgagaggtggctgacggcgccgaggagcccctcagaacctgcaggtggggggagctcccatatcttacgggtgtttcggg	
Primers	
Forward primer	5?-gtcttcactcccgtgcttgt-3?
Reverse primer	5?-cccgaaacacccgtaagata-3?
Sequencing primer	5?-acctctcacaccccctcac-3?
**Amplification of pri-mir-149 and pre-mir-149 for assaying expression**	
Amplicon	
cctcgatccagcctgcccgaggctcccaggccttcgcccgccttgcgtccagcctgccgggggctcccaggccggcgcccgagctctggctccgtgtcttcactcccgtgcttgtccgaggagggagggagggacgggggctgtgctggggcagc(c)ggaacaacgcaggtcgccgggccggctgggcgagttggccgggcggggctgaggggtcggcgggggaggctgaggcgcgggggccggtgcgcggccgtgagggggtgtgagaggtggct	
Primers	
Pri-mir-149 forward primer	5?-gccttgcgtccagcct **-**3?
Pri-mir-149 reverse primer	5?-cccccgtccctccct-3?
Pre-mir-149 forward primer	5?-ctggctccgtgtcttcact-3?
Pre-mir-149 reverse primer	5?-cccccgtccctccct-3?

(c): Polymorphism site.

### Study Subjects

The study subjects consisted of 122 controls and 273 HNSCC patients. They were recruited from the Mackey Memorial Hospital and the National Yang-Ming University Hospital. The clinicopathological data is presented in Table 1. Among these study subjects, data related to BQ chewing and tobacco smoking are recorded. However, the status of viral infection was not determined and the limitation of this study remained. The control individuals included 62 females and 60 males. They were individuals with diagnosis other than cancer and admitted due to surgery for benign oral lesions, trauma management, pre-prosthetic surgery and removal of impacted teeth. Blood samples were draw pre-operatively from these subjects. Tissue specimens were taken from 70 HNSCC tumors, along with paired non-cancerous matched tissue (NCMT) samples. Among the tissues pairs, 24 pairs were from buccal mucosa, 23 pairs from tongue, 10 pairs from gingiva, 3 pairs from retromolar trigone, 2 pairs from floor of mouth, 2 pairs from lip and 6 pairs from oropharyngeal region. There were also 24 neck lymph node metastatic lesions paired with corresponding primary HNSCC available for analysis. Tissues were stored at −80°C until use. Frozen sections were prepared for microdissection to retrieve pure epithelial cells for analysis [14]. A fraction of each tissue sample was also fixed in formalin and processed to give paraffin sections.

**Figure 1:**
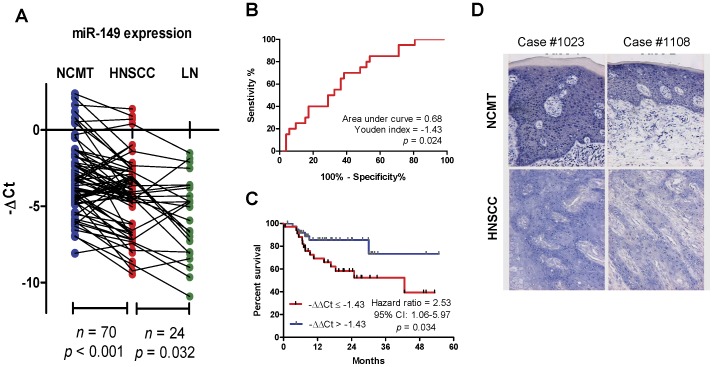
*miR-149* expression in HNSCC tissues. (A) Before-and-after plots of the −ΔCt values from paired NCMT tissue, primary HNSCC and neck lymph node metastasis (LN). Paired *t*-test. (B) ROC analysis across the –ΔΔCt values from survived patients and dead patients. (C) Kaplan-Meier analysis of overall survival as related to *miR-149* expression using −ΔΔCt = −1.43 as a cutoff. (D) ISH. Upper, NCMT, lower HNSCC. Note the *miR-149* signals in nucleus and cytosol. NCMT exhibited *miR-149* signals that were more intense than HNSCC.

**Figure 2:**
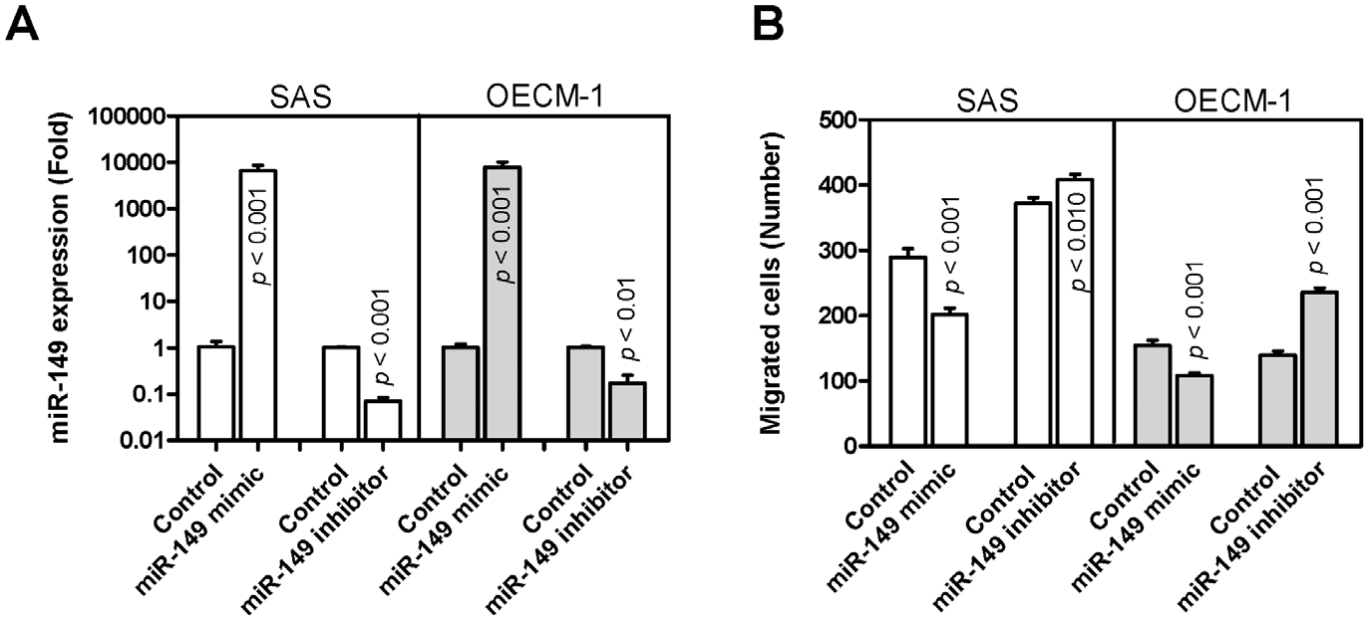
*miR-149* expression and HNSCC cell migration. SAS and OECM-1 cells were transfected with *miR-149* mimic and *miR-149* inhibitor. (A) qRT-PCR analysis. This detected increased *miR-149* expression and decreased *miR-149* expression following transfecting with *miR-149* mimic and *miR-149* inhibitor, respectively, relative to the controls. (B) Transwell migration assay. This indicated that transient *miR-149* expression decreased cell migration, and knockdown of *miR-149* expression increased cell migration. The results are means ± SE from at least triplicate analysis; un-paired *t*-test.

### DNA Extraction

DNA was prepared from peripheral leukocytes using a genomic DNA extraction kit QIAamp™ DNA Mini Kit (QIAGEN, Valencia, CA). The extraction procedure followed a previously described protocol [20]. The concentration of purified genomic DNA was in the range 4 ng/µl to 12 ng/µl.

**Figure 3:**
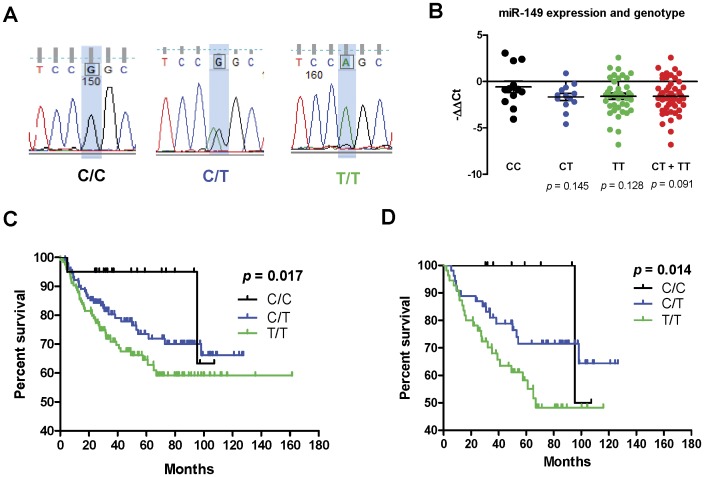
Analysis of the pre-*mir-149* polymorphism. (A) Sequencing analysis. Representative sequencing results showing the genotypes of pre-*mir-149* polymorphisms using blood DNA. (B) Scatter spot charts to show the levels of *miR-149* expression in HNSCC patients with various genotypes. The comparison was made against the C/C genotype. Un-paired *t*-test. (C, D) Kaplan-Meier analysis of overall survival in all HNSCC (C) and HNSCC occurring on buccal mucosa (D).

**Table 3:** HNSCC susceptibility as related to pre-*mir-149* polymorphism (univariate analysis).

HNSCC susceptibility and pre-mir-149 polymorphism					
Genotype	C/C	C/T		T/T	*p* value
Control	21	52		49	0.012*
HNSCC	20	129		124	
**Allelotype**	**C**		**T**		***p*** ** value**
Control	94 (38.5%)		150 (61.5%)		0.041#
HNSCC	169 (31.0%)		377 (69.0%)		

* Chi-square analysis.

# Fisher’s exact test.

**Table 4:** HNSCC susceptibility as related to pre-*mir-149* polymorphism (multivariate analysis).

Logistic regression model for HNSCC susceptibility (dominant model)#			
Variables	OR	95% CI	*p* value
Genotype: T/T *vs.* C/C+C/T	0.91	0.51 to 1.61	0.748
Age	1.00	0.98 to 1.02	0.968
Gender: Male *vs.* Female	6.98	3.18 to 15.31	<0.001
BQ chewer *vs.* Non-BQ chewer	0.93	0.43 to 2.00	0.850
Smoker *vs.* Non-smoker	2.63	1.20 to 5.78	0.016
**Logistic regression model for HNSCC susceptibility (recessive model)** #			
**Variables**	**OR**	**95% CI**	***p*** ** value**
Genotype: T/T+C/T *vs.* C/C	2.37	0.97 to 5.80	0.059
Age	1.00	0.97 to 1.02	0.710
Gender: Male *vs.* Female	6.50	2.96 to 14.26	<0.001
BQ chewer *vs.* Non-BQ chewer	0.84	0.39 to 1.83	0.669
Smoker *vs.* Non-smoker	2.57	1.16 to 5.67	0.020

OR, odds ratio; CI, confidence interval.

# All predictor variables are adjusted.

### Genotyping

The 248-bp sequence encompassing pre-*mir-149* was downloaded from a NCBI blast search (http://blast.ncbi.nlm.nih.gov/Blast.cgi) and was amplified using a forward primer (5′-gtcttcactcccgtgcttgt-3′) and a reverse primer (5′-cccgaaacacccgtaagata-3′). Genotyping was performed by direct sequencing of the amplicons (Table 2). The high-throughput genomic analysis was carried out using the thermal cycle sequencing method and a 96-capillary 3730XL DNA analyzer (Applied Biosystems, Foster City, CA). Restriction fragment length polymorphism (RFLP) assay was also used to confirm the results of some cases when there was questionable sequencing result. The *PvuII* restriction enzyme was utilized to recognize the CAGCTG sequence located within polymorphism. Analysis of the T/T homozygous allele yields 62-bp and 186-bp bands after digestion with *PvuII.* However, *PvuII* is unable to digest the C/C homozygous allele and only the undigested 248-bp fragment is found after *PvuII* digestion.

**Table 5:** Clinicopathological variables as related to pre-*mir-149* polymorphism.

Variables	C/C	C/T	T/T	*p* value
T1–3	15	72	50	0.003
T4	5	57	74	
N0	15	93	65	0.003
N+	5	36	59	
Stage I–III	14	63	44	0.006
Stage IV	6	66	80	
Perineural invasion (−)	16	114	114	0.241
Perineural invasion (+)	4	15	10	
Lymphovascular invasion (−)	16	116	114	0.503
Lymphovascular invasion (+)	4	13	10	

Chi-square analysis.

### qRT-PCR Analysis for *miR-149*

A TaqMan miRNA assay kit and supplies were used to quantify the expression of *miR-149* and miR-149* according to the manufacturer’s instructions (Applied Biosystems). *RNU6B* and *Let-7a* were used as internal controls [14]. Ct was the number of cycles at which the fluorescence signal passed the threshold. −ΔCt was the difference in Ct values between the internal controls and *miR-149*. −ΔΔCt was the difference in ΔCt values between the experimental settings or samples. 2^−ΔΔCt^ represents the fold change in *miR-149* expression.

### qRT-PCR for Pri*-mir-149* and Pre*-mir-149*

The expression of pri-*mir-149* and pre-*mir-149* were also determined by TaqMan qRT-PCR analysis. The primer sequences are described in Table 2. The rs2292832 polymorphism is localized outside the pri-*mir-149* and pre-*mir-149* amplicons. The TaqMan probes were synthesized by Ambion (Carlsbad, CA) and the specificity of the quantitative analysis was tested using synthetic oligonucleotide mimics of pri-*mir-149* and pre-*mir-149*.

**Table 6:** Multivariate analysis for the prediction of neck nodal metastasis in HNSCC.

Logistic regression for prediction of neck nodal metastasis#				
Variables	Subgroups (*n*)	OR	95% CI	*p* value
pre-*mir-149* polymorphism	T/T (124) *vs.* C/C+C/T (149)	2.81	1.58 to 4.97	<0.001
Age		0.99	0.97 to 1.02	0.636
Gender	Male (251) *vs.* Female (22)	6.52	1.31 to 32.50	0.022
Tumor size	T4 (136) *vs*. T1–3 (137)	2.14	1.20 to 3.82	0.010
BQ chewer	Yes (236) vs. No (37)	0.92	0.33 to 2.55	0.867
Smoker	Yes (246) vs. No (27)	0.60	0.22 to 1.62	0.310
Perineural invasion	Positive (29) *vs*. Negative (244)	1.12	0.35 to 3.53	0.853
Lymphovascular invasion	Positive (27) *vs.* Negative (246)	19.47	4.91 to 77.28	<0.001

OR, odds ratio; CI, confidence interval.

# All predictor variables are adjusted.

**Table 7:** Multivariate analysis for survival in HNSCC.

Cox-proportional hazard model for prediction of patient’s survival#				
Variables	Subgroups (*n*)	HR	95% CI	*p* value
pre-*mir-149* polymorphism*	T/T (124) *vs*. C/C+C/T (149)	1.66	1.05 to 2.60	0.030
Age		1.01	0.99 to 1.03	0.387
Gender	Male (251) *vs.* Female (22)	0.76	0.22 to 2.64	0.664
BQ chewer	Yes (236) *vs.* No (37)	1.21	0.47 to 3.13	0.699
Smoker	Yes (246) *vs.* No (27)	2.08	0.72 to 6.01	0.180
Perineural invasion	Positive (29) *vs*. Negative (244)	1.99	0.94 to 4.24	0.075
Lymphovascular invasion	Positive (27) *vs.* Negative (246)	2.94	1.43 to 6.04	0.003

HR, hazard ratio; CI, confidence interval.

# All predictor variables are adjusted.

* The impact of the polymorphism on survival is no longer significant when adjusting for tumor size or nodal status.

**Table 8:** Multivariate analysis for the prediction of neck nodal metastasis in buccal mucosa carcinoma.

Logistic regression for prediction of neck nodal metastasis#				
Variables	Subgroups (*n)*	OR	95% CI	*p* value
pre-*mir-149* polymorphism	T/T(56) *vs.* C/C+C/T (63)	2.73	1.12 to 6.62	0.027
Age		0.98	0.94 to 1.03	0.386
Gender	Male (114) *vs.* Female (5)	4.60	0.09 to 242.21	0.451
Tumor size	T4 (58) *vs*. T1–3 (61)	4.01	1.58 to 10.17	0.003
BQ chewer	Yes (109) vs. No (10)	0.35	0.02 to 6.79	0.485
Smoker	Yes (106) vs. No (13)	0.30	0.04 to 2.25	0.242
Perineural invasion	Positive (6) *vs*. Negative (113)	0.00	0.00 to 0.00	0.995
Lymphovascular invasion	Positive (7) *vs.*Negative (112)	0.00	0.00 to 0.00	0.994

OR, odds ratio; CI, confidence interval.

# All predictor variables are adjusted.

**Table 9:** Multivariate analysis for survival in buccal mucosa carcinoma.

Cox-proportional hazard model for prediction of patient’s survival#				
Variables	Subgroups (*n*)	HR	95% CI	*p* value
pre-*mir-149* polymorphism*	T/T(56) *vs.* C/C+C/T (63)	2.19	1.13 to 4.24	0.021
Age		1.01	0.98 to 1.05	0.516
Gender	Male (114) *vs.* Female (5)	1.95	0.12 to 30.84	0.638
BQ chewer	Yes (109) vs. No (10)	0.45	0.08 to 2.46	0.361
Smoker	Yes (106) vs. No (13)	2.09	0.34 to 12.91	0.428
Perineural invasion	Positive (6) *vs*. Negative (113)	4.59	0.60 to 35.34	0.146
Lymphovascular invasion	Positive (7) *vs.*Negative (112)	0.48	0.05 to 4.74	0.529

HR, hazard ratio; CI, confidence interval.

# All predictor variables are adjusted.

* The impact of the polymorphism on survival is no longer significant when adjusting for tumor size or nodal status.

### Cell Culture, Transient Expression and Knockdown of Expression

HNSCC cell lines, including Fadu, OECM-1 and SAS, as well as 293FT, were cultured as previously described [14], [27]. Chemically modified *miR-149* mimic (Applied Biosystems) was used for transient *miR-149* expression. *miR-149* inhibitor (Applied Biosystems) was used to knockdown *miR-149* expression. The controls for the transient expression and knockdown of *miR-149* were also purchased from Applied Biosystems. TransFectin™ Lipid Reagent (BioRad Lab, Hercules, CA) was the transfection reagent.

**Figure 4:**
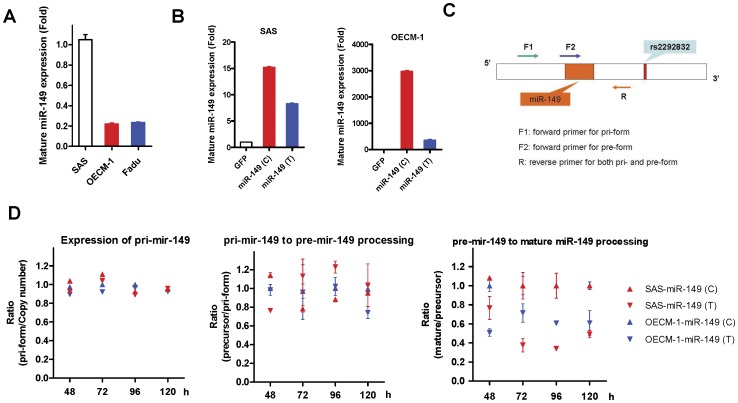
Analysis of *miR-149* expression in HNSCC subclones. (A) *miR-149* expression in SAS, Fadu and OECM-1 cells carrying the C/T, T/T and T/T phenotypes, respectively. (B) *miR-149* expression in the SAS-GFP, SAS-*miR-149*(C) and SAS-*miR-149*(T) subclones (Lt); and OECM-1-GFP, OECM-1-*miR-149*(C) and OECM-1-*miR-149*(T) subclones (Rt). T variant seemed to be associated with decreased *miR-149* expression. Data shown are the means ± SE from triplicate analysis. (C) Schematic diagram to illustrate the analytical strategies used to quantify the expression of the pri-*mir-149*, pre-*mir-149* and *miR-149* in subclones. (D) Processing efficiency at different time points from 48 h to 120 h. Lt, processing of pri-*mir-149* (normalized against viral integration copy revealed by Late-RT qPCR); Middle, pri-*mir-149* to pre-*mir-149* processing; Rt, pre-*mir-149* to mature *miR-149* processing. Both SAS and OECM-1 unequivocally display a decrease in pre-*mir-149* to *miR-149* processing in the T variant subclones. All ratios (Y axis) were normalized against the OECM-1-*miR-149*(C) ratio at the same time point. Data shown are the means ± SE from duplicate analysis.

### Transwell Migration Assay

Cells were grown in media containing 0.5% fetal bovine serum (FBS, Biological Industries, Kibbutz Beit Haemek, Israel) on a Transwell system (Corning, Acton, MA) with a membrane pore size of 8 µm. Cell migration was induced by the addition of 10% FBS to the culture medium in lower chamber to form a serum gradient. The cell growth was arrested by treatment with 1 µM hydroxyurea (Sigma-Aldrich, St Louise, MO). The migrated cells on the lower surface were counted by fluorescence microscopy after staining with Hoechst 33258 (Sigma-Aldrich).

### *In situ* Hybridization (ISH)

miRCURY LNA™ *miR-149* probe labeled with digoxigenin and all associated reagents were purchased from Exiqon (Vedbaek, Denmark). After re-hydration, the paraffin sections were digested by protease K and fixed with 4% paraformaldehyde. Sections were then pre-hybridized for 4 h, and hybridized with the 10 µM *miR-149* probe at 45°C overnight. Slides were washed, and then incubated with anti-digoxigenin antibody at room temperature for 3 h. NBT/BCIP (5-bromo-4-chloro-3-indolyl-phosphate and nitroblue tetrazolium) solution reagent was used to detect the signal.

### Plasmid Construction, Viral Infection and Late-RT-qPCR

For exogenous *miR-149* expression, pri-*mir-149* (sequence marked by arrows in Table 2) with either the T or C genotypic variant was cloned into a lentivirus vector, pLV-EF1a-GFP, which contains a green fluorescent protein (GFP) tag. An empty vector or a vector carrying the inserts were co-transfected with helper plasmids into 293FT cells to produce lentivirus and then stable cell lines were established by lentiviral infection. Late-RT-qPCR to detect reverse transcription of the lentivirus was carried out to identify the genomic integration of viral sequence as a late event of viral infection [28].

### Statistical Analysis

The Hardy–Weinberg equilibrium (HWE) was calculated for controls to exclude sampling bias. The last date of follow-up was either the date of death or the last date the patient was contacted. Overall survival was estimated using the Kaplan–Meier analysis, with comparisons between groups made by the log-rank test. The cutoff value for *miR-149* was determined using the Youden index obtained from the receiver operating characteristic (ROC) curve [14]. The relationship between clinicopathological variables and genotyping was analyzed by Fisher’s exact test or chi-square analysis. The difference between experiment groups or sample groups was analyzed with *t*-test. Logistic regression was performed to adjust the relevant covariates of neck lymph node metastases including tumor size, perineural invasion and lymphovascular invasion to achieve an adjusted odds ratio (OR) and 95% confidence interval (95% CI). Cox proportional hazards regression model was performed to determine potential prognostic factors for survival, the hazard ratio (HR) and 95% CI. Differences between values were considered significant when a two-tailed *p* was <0.05. Statistical analysis was performed with Prism 5 (Graph-Pad, San Diego, CA) or SPSS 17.0 (SPSS Inc., Chicago, IL) software.

## Results

### Down-regulation of *miR-149* Expression in HNSCC Tissues

The expression of *miR-149* in HNSCC and paired NCMT was determined by qRT-PCR analysis. Paired-*t*-test indicated a significant down-regulation of *miR-149* expression in HNSCC tissue relative to the paired NCMT (*p*<0.001) (Fig. 1A). Moreover, statistically significant difference in *miR-149* expression was noted between primary HNSCC and their paired metastatic lesions (*p* = 0.032). ROC analysis validated a significant separation of the patient’s survival status when using a –ΔΔCt cutoff value of −1.43 (Fig. 1B). In addition, tumors having a −ΔΔCt ≤−1.43 exhibited a significantly worse prognosis than contrasting group (Fig. 1C). To view the *miR-149* transcript in tissue samples, ISH was performed on six NCMT/HNSCC pairs. A representative illustration of two tissue pairs are shown in Fig. 1D and these show strong blue staining in the nucleus and cytosol in epithelial cells of the NCMT. However, the blue staining in the nucleus and cytosol of the tumor cells is less intensive than that in matched NCMT (Fig. 1D). Since the other four tissue pairs exhibited similar staining pattern, down-regulation of *miR-149* expression in HNSCC tissue does seem to be confirmed by ISH.

### *miR-149* Expression is Associated with a Decreased Migration of HNSCC Cells

Exogenous expression of *miR-149* was carried out by the transfection of *miR-149* mimic. Such treatment increased *miR-149* expression by >1000-fold in both SAS and OECM-1 cells (Fig. 2A). Knockdown of *miR-149* expression by transfection with the *miR-149* inhibitor decreased the expression to one-tenth to one-third level of the controls using HNSCC cells. After transient expression or knockdown of expression, the proliferation of HNSCC cells was not affected (not shown). However, transient *miR-149* expression decreased and knockdown of *miR-149* expression increased the migration of both SAS and OECM-1 cells (Fig. 2B).

### Genotype and HNSCC Risk

Since *miR-149* expression is able to suppress HNSCC cell mobility and this should affect patient survival through regulating invasion or metastasis, the presence of the rs2292832 polymorphism in pre-*mir-149* across a non-cancer control cohort and a cancer patient cohort was analyzed. Genotyping by direct sequencing of amplicons was carried out. The analysis is shown in Fig. 3A. There were 15 subjects being additionally validated with RFLP to resolve questionable sequencing data. The genotypic distribution of 122 healthy controls fulfills the HWE (p^2^+2pq+q^2^ = 1: chi-square = 0.653, *p* = 0.722). The genotypic distribution of 273 HNSCC patients also fulfills the HWE (chi-square = 1.510, *p* = 0.470). These examinations may exclude the sampling bias (Table 3). In univariate analysis, there was significantly higher frequency of the T/T genotype and the T allele in HNSCC patients compared to controls, which suggest a potential association between the pre-*mir-149* polymorphism and HNSCC susceptibility (Table 3 and 4). Dominant and recessive models for multivariate analysis were carried out. HNSCC susceptibility was unequivocally shown associated with gender and smoking status in both models. Analysis using dominant model analysis did not reveal an association between T/T genotype and HNSCC susceptibility. Although analysis using recessive model revealed a protective trend of C/C genotype for HNSCC susceptibility, the statistical difference was not significant (Table 4). This polymorphism may not be associated with HNSCC susceptibility.

### The T/T Genotype is Associated with HNSCC Progression

The relationship between clinicopathological variables and genotypes in HNSCC subjects was further analyzed. The results showed that the proportion of patients with the T/T genotype is significantly higher than that of the other genotypes among the more advanced disease groups, including larger tumors, tumors with neck nodal involvement, and stage IV patients (Table 5). Nodal status is the most important clinical event correlated with pre-*mir-149* polymorphism. This polymorphism was not associated with other variables. In HNSCC cases receiving both expression analysis and genotyping, subjects carrying the T/T and/or C/T genotypes tended to have lower *miR-149* expression relative to subjects carrying the C/C genotype, however, the difference is not statistically significant (Fig. 3B).

### The T/T Genotype is Associated with Nodal Metastasis and a Poor Prognosis Among HNSCC Patients

Kaplan-Meyer analysis indicated that patients carrying T/T genotype had worse prognosis than other genotypes (Fig. 3C). Multivariate modules were used to specify the power of the T/T genotype when predicting nodal metastasis and patient survival by adjusting various cofounding factors. Logistic regression analysis indicated that the T/T genotype was an independent factor when predicting nodal metastasis with an odds ratio of 2.81 (95% CI: 1.58 to 4.97) (Table 6). Cox-proportional hazard model identified that the T/T genotype was a risk factor to predict patient survival with a hazard ratio of 1.66 (95% CI: 1.05 to 2.60) (Table 7). In addition, lymphovascular invasion were found to predict both nodal metastasis and patient’s survival. However, the impact of this polymorphism on survival no longer exists when adjusting for tumor size or nodal status (detailed analysis not shown), implicating that the impact of the polymorphism on survival is mediated by its effects on metastasis (Table 6). As buccal mucosa carcinoma subset of HNSCC is highly associated with endemic BQ chewing in Asians [11], [12], it is also the most frequent HNSCC subset in our samples (*n* = 119; Table 1). Dissection further identified that T/T genotype was associated with the worst prognosis of buccal mucosa carcinoma (Fig. 3D). However, this polymorphism was not associated with HNSCC localized at non-buccal mucosa regions. Logistic regression specified that T/T genotype predicted the nodal metastasis (odds ratio of 2.73; 95% CI: 1.12 to 6.62) (Table 8). Cox-proportional hazard model identified that the T/T genotype also predicted patient survival (hazard ratio of 2.19; 95% CI: 1.13 to 4.24) (Table 9). Similarly, the influence of the polymorphism on survival is also mediated by its effects on nodal metastasis.

### The Expression Level of *miR-149* is Associated with the Pre*-mir-149* Polymorphism

SAS, Fadu and OECM-1 cells carry C/T, T/T and T/T genotype, respectively. *miR-149* expression in SAS cells was higher than in Fadu and OECM-1 cells (Fig. 4A). To address if the pre-*mir-149* polymorphism may underlie differential *miR-149* expression, pre-*mir-149* sequences with either the C variant or the T variant were amplified, cloned into a vector to produce lentivirus and then infected into SAS and OECM-1 cells to achieve stable subclones (Table 2), which were designated SAS-GFP, SAS-*miR-149*(C), SAS-*miR-149*(T), OECM-1-GFP, OECM-1-*miR-149*(C), and OECM-1-*miR-149*(T). Interestingly, SAS-*miR-149*(C) and OECM-1-*miR-149*(C) exhibited higher *miR-149* expression than SAS-*miR-149*(T) and OECM-1-*miR-149*(T) (Fig. 4B). It has been reported that genotypic variation in the precursor region might influence the processing of mature miRNA [18]. Thus an approach was designed to quantify the integrated copy number of the various lentiviruses together with the expression of pri-*mir-149*, pre-*mir-149* and *miR-149* in the subclones (Fig. 4C). Quantification was performed on cells at different confluence after seeding for 48 h to 120 h. The ratio of pri-*mir-149* to viral integration copy was similar across the subclones at different time point (Fig. 4D, Lt). The ratio of pri-*mir-149* to pre-*mir-149* seemed to slightly fluctuate at different time points. Whereas, no consistent difference was observed between clones carrying the C variant and T variant (Fig. 4D, middle). However, the ratio of mature *miR-149* to pre-*mir-149* in SAS-*miR-149* (T) and OECM-1-*miR-149* (T) subclones were consistently lowered than the (C) subclones (Fig. 4D, Rt). These findings suggest that the T variant in pre-*mir-149* may hinder the processing of pre-*mir-149* to mature *miR-149*, which then changes the abundance of *miR-149* in HNSCC cells.

## Discussion

Previous studies have shown that *miR-149* is down-regulated in oral carcinoma and astrocytoma [5], [14], [24]. This is the first study identify its down-regulation in HNSCC and link this down-regulation to a worse patient survival. qRT-PCR analysis performed on microdissected tissues confirmed the down-regulation of the mature form of *miR-149* in tumor cells compared to adjacent non-cancerous epithelial cells; furthermore, the ISH assays also confirmed these findings. Although ISH is a powerful tool that allows the detection of the location and amount of a transcript, it was not used as a standard assay in this study as it also is able to detect nuclear signals of pri-*mir-149* [29]. The down-regulation of *miR-149* expression from primary HNSCC to metastatic lesion further indicates the roles of *miR-149* for HNSCC progression. In this study, we delivered *miR-149* mimic duplex that had been modified to ensure the right use of the guide strand of this duplex in RISC. *miR-149* expression was found to suppress tumor cell migration. The various phenotypic influences were also confirmed by knockdown experiments. Recent reports have indicated that the passenger strand of the *miR-149* duplex, miR-149*, acts as a distinct miRNA that exerted a wide variety of apoptotic regulation effects on in cancer cells [30], [31]; therefore, our approach strategy has been to ascertain the impact of *miR-149* on cell migration, and in the process bypass any effect from miR-149*. Since the processing of pre-*mir-149* through lentiviral delivery will yield a duplex of both *miR-149* and miR-149*, phenotypic assays were not performed on the stable subclones. Up to the present, no gene has been reported to be the target of *miR-149* and therefore our preliminary assays were unable to validate any gene as a true *miR-149* target in HNSCC cells. Further investigations are required to discover any genes that are negatively regulated by *miR-149*, especially those that are able to promote cell mobility.

Genetic variants in miRNA sequences that might drive differential functional impacts have attracted attention recently [32]. Polymorphism in pre-*mir-196a2* has been reported to increase the risk of colorectal carcinoma, hepatocellular carcinoma and HNSCC [19], [33]. Polymorphism in pre-*mir-146a* has been shown to be a risk factor for breast carcinoma [34], gastric carcinoma [35] and oral carcinoma [36]. rs2292832 polymorphism in pre-*mir-149* has been reported to be correlated with susceptibility to gastrointestinal carcinoma, with C/T and C/C individuals showing a protective effect [21]. However, this polymorphism was not correlated with the breast carcinoma and non-small cell lung carcinoma (NSCLC) susceptibility [23], [37]. The current study shows that the pre-*mir-149* polymorphism is not associated with susceptibility of HNSCC in our study cohort. Similar result was also reported in non-Hispanic HNSCC population [22]. Polymorphisms in pre-*mir-196a2* or pre-*mir-146a* have also been associated with poor prognosis of NSCLC and oral carcinoma [18], [20]. This is also the first study to show that T/T HNSCC patients seem to have more advanced tumor progression and a poorer prognosis. Multivariate analysis stratified the T/T genotype as an independent predictor and therefore it is a possible biomarker for HNSCC progression. For HNSCC occurring on oropharynx, the presence of HPV in tumor samples is strongly associated with patient’s survival [38]. Our assay identified the association between at T/T genotype in pre-*mir-149* and the poor survival of HNSCC occurring on buccal mucosa. Several molecular markers were found to be prognostic predictors of buccal mucosa carcinoma [12], combined use of miR-149 polymorphism with these markers in future could validate more powerful prognostic prediction for buccal mucosa carcinoma [11]. Just as the transient *miR-149* expression ablates the mobility of tumor cells, the induction of *miR-149* may have a therapeutic efficacy against HNSCC progression. Since the C allelic frequency was only 0.31 in HNSCC patients, and therefore C/C tumor samples were few, we were unable to confirm that T/T tumor samples had lower level of *miR-149* expression in this study. Nevertheless, validation of pre-*mir-149* polymorphism as biomarker using blood samples may be useful in a preventive capacity and should also help with tumor prognosis assessment; in the latter case the approach is simpler than complicated tumor tissue analysis.

Polymorphisms occurring in the core region of a miRNA would be expected to influence the translation repression of miRNA due to its altered mRNA binding affinity following sequence change [17]. Polymorphism in the promoter region of a miRNA would be expected to affect the synthesis of miRNA and the changes in expression of the miRNA would then indirectly impairs target gene expression subsequently [39]. However, the functional implications of polymorphisms occurring in either the primary or precursor form of a miRNA remain largely unclear. We postulate that this type of genetic variant may influence the processing of the mature miRNA [18]. Experiments have identified that, relative to the C variant, the T variant in precursor form may slow down the processing from pre*-mir-149* to mature *miR-149*. The result would be a decrease in *miR-149* that, in turn, may attenuate the suppression effect of *miR-149* on cell motility. This type of change may underlie the effect of *miR-149* on the susceptibility and progression of HNSCC. Although the actual mechanism still needs further elucidation, potentially the RNA binding proteins involved in miRNA processing may contribute to this difference. The RNA binding protein Lin28 inhibits the processing of *let-7* family by uridylation of the 3′ nucleotides of the precursor, which acts as a obstacle to Dicer processing; the uridylated precursor then undergoes degradation [40], [41]. Moreover, the enzyme, TUT4, which is also responsible for precursor uridylation, is able to recognize the sequence GGAG and in concert with Lin28 interferes with the processing of multiple miRNAs [42]. Three GGAG sequences are present in the precursor region of *miR-149*. However, since the polymorphism site is not within these sequences, the role of Lin28 in *miR-149* processing needs further investigation.

This study demonstrated the down-regulation of *miR-149* expression is associated with increased tumor cell mobility in HNSCC. The T/T genotype of the pre-*mir-149* polymorphism may contribute to the down-regulation of expression, and this, in turn, seems to determine the risk of HNSCC and prognosis of HNSCC patients.
